# Oscillometric arterial blood pressure in haemodynamically stable neonates in the first 2 weeks of life

**DOI:** 10.1007/s00467-023-05979-x

**Published:** 2023-05-05

**Authors:** Judit Klara Kiss, Anna Gajda, Judit Mari, Judit Nemeth, Csaba Bereczki

**Affiliations:** Department of Paediatrics, University of Szeged, Szeged, 6720 Hungary

**Keywords:** Neonate, Normal blood pressure, Blood pressure percentile, Birth weight, Gestational age

## Abstract

**Background:**

We aimed to provide data on the normal blood pressure of haemodynamically stable neonates. Our study uses retrospective, real-life oscillometric blood pressure measurement values to determine the expected blood pressure in different gestational age, chronological age and birth weight groups. We also investigated the effect of antenatal steroid on neonatal blood pressure.

**Methods:**

Our retrospective study (2019–2021) was carried out in the Neonatal Intensive Care Unit of the University of Szeged, Hungary. We involved 629 haemodynamically stable patients and analysed 134,938 blood pressure values. Data were collected from electronic hospital records of IntelliSpace Critical Care Anesthesia by Phillips. We used the PDAnalyser program for data handling and the IBM SPSS program for statistical analysis.

**Results:**

We found a significant difference between the blood pressure of each gestational age group in the first 14 days of life. The systolic, diastolic and mean blood pressure rise are steeper in the preterm group than in the term group in the first 3 days of life. No significant blood pressure differences were found between the group with a complete antenatal steroid course and those who received incomplete steroid prophylaxis or did not receive antenatal steroids.

**Conclusion:**

We determined the average blood pressure of stable neonates and obtained normative data by percentiles. Our study provides additional data on how blood pressure varies with gestational age and birth weight.

**Graphical abstract:**

**Figure:**
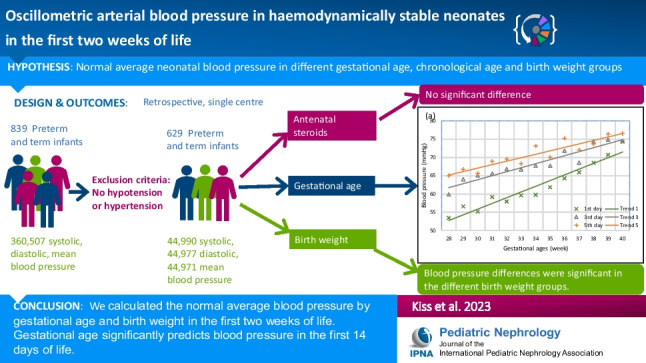
A higher resolution version of the Graphical abstract is available as Supplementary information

**Supplementary information:**

The online version contains supplementary material available at 10.1007/s00467-023-05979-x.

## Introduction

Blood pressure measurements are essential to evaluate the cardiovascular stability of preterm and term infants. Patients who have received neonatal intensive care unit (NICU) treatments have a well-recognised risk of developing hypertension, affecting their long-term cardiovascular and kidney health [1, 2]. Diagnosing and treating hypotension and hypertension are important to reduce the lifelong consequences of prematurity. Over the last 40 years, several studies have provided data on the blood pressure values of preterm and term NICU patients [3–11]. However, defining normal blood pressure remains very challenging in this population. The difficulties arise from the different measurement methods, patient population and wide variety of prenatal and postnatal influencing factors examined by different studies [3–11]. Additionally, one of the greatest issues in defining the stable patient group is selecting the interventions that are considered routine in neonatal care.

Gestational age and birth weight are known factors affecting the blood pressure of newborn infants. Several studies investigated neonatal blood pressure and its influencing factors related to maternal and infant health and therapy, but the results are still controversial [4, 8, 9, 12, 22].

The intra-arterial blood pressure measurement method is still considered the gold standard in the evaluation of neonatal blood pressure. However, arterial lines are associated with several side effects, making them less frequently used in non-critically ill neonatal patients.

Our study provides and evaluates a large amount of data on oscillometric blood pressure values in hemodynamically stable NICU patients intending to determine the normal average blood pressure in patients with different gestational ages and birth weights.

## Methods

Our retrospective single-centre study was performed at the NICU of the University of Szeged, Hungary. Over a 3-year period (from 01 January 2019 to 31 December 2021), all the blood pressure readings were collected from the entire patient group admitted to the unit.

In order to select haemodynamically stable neonates, we excluded all infants with a substantial risk of hypotension or hypertension from the study. The exclusion criteria [4–6, 18] were the following: (1) need for inotropic support; (2) postnatal steroid administration; (3) patients who required invasive ventilation for more than 24 h; (4) renal parenchymal disease, renovascular abnormality and acute kidney injury; (5) major congenital heart defect; (6) chromosomal anomaly; (7) intracranial hypertension; (8) diagnosed hypertension of the newborn; (9) maternal substance abuse and withdrawal syndrome of the newborn; (10) use of an umbilical arterial catheter; (11) grades III and II bronchopulmonary dysplasia (BPD); (12) endocrine disorder with a risk of hypertension and (13) death. Newborns with mild BPD who were not diagnosed with hypertension during their NICU stay were included in the study population since, in most BPD patients, hypertension starts after discharge from the NICU [13]. As there have been changes in neonatal medicine since the earlier research, we have not excluded interventions that are part of routine care today. Therefore, patients ventilated on nasal CPAP, who received caffeine or who had short-term procedural analgesia, were involved in the study. The neonates who received diuretics without the diagnosis of hypertension were included in our clinical investigation. We also collected maternal medical data. However, we did not exclude patients based on the investigated maternal medical problems, as the results are still controversial on their effect on neonatal blood pressure [12]. Over the study period, the unit admitted 839 patients. After applying the exclusion criteria, we collected blood pressure data from 629 preterm and term infants for analysis in the study.

The blood pressure values and patient and maternal demographic and medical data were retrieved from the IntelliSpace Critical Care Anesthesia (ICCA) electronic medical records by Phillips and the hospital information system, Medsol. The collected data included all the blood pressure records, antenatal steroids, Apgar scores at 1 and 5 min of life, birth weight, ventilated days (either invasive or non-invasive), neonatal medication (caffeine citrate, diuretics, ibuprofen, analgesia), maternal medical history (hypertension, gestational diabetes mellitus, insulin-dependent diabetes mellitus, smoking) and neonatal diagnosis (intraventricular haemorrhage, patent ductus arteriosus, BPD, necrotising enterocolitis, sepsis).

The gestational age was determined with the use of the last menstrual period and early ultrasound scans by the obstetrics team. The obstetrics team applied a complete steroid course, which means four times 6 mg doses of dexamethasone administered intramuscularly every 12 h [23]. The steroid course was considered incomplete when the suggested dose was not entirely given.

We calculated the weight percentiles with the use of Fenton growth charts. Blood pressures were measured according to the unit’s existing blood pressure measurement and hypertension guidelines. Generally, a single blood pressure measurement was taken. On a daily average, the number of measures by patient varied between one and twelve in the study group’s first two weeks of life. The medical team decided the frequency of the measurements based on the patient’s condition. The aim was to avoid repeating measurements to ensure comfort and keep the settled states of the neonates under the measure. In case of invalid values or suspected hypo- or hypertension, the blood pressure was remeasured based on the medical team’s decision. The unit used the blood pressure percentile table created by Dionne et al. [5] for the diagnosis of hypertension. Diagnosis of hypotension was based on the blood pressure results published by Kent, Pejovic and Zubrow [4, 6, 8].

Blood pressure was measured using an oscillometric device with an appropriate-sized cuff (cuff width to the arm circumference ratio closest to 0.50) [14, 15]. We preferably used the right upper arm to measure the blood pressure. Calf blood pressure measurements were used in case of contraindications such as right arm tissue injury, peripheral cannula or PICC-line [16–18]. According to the literature, calf blood pressure can be used during the first 6 months of life as the results are similar to upper limb blood pressure measurements [18]. Patients with a congenital heart defect (e.g. coarctation of aortae) were excluded from the patient group.

Data on the infants’ wakefulness state were unavailable in the medical records. According to our protocol, the measurements were done during sleep or in a quiet awake state. In case of an unsuccessful measurement (e.g. the newborn woke up during measurement and cried), the protocol was to remeasure the blood pressure in a settled state.

**Fig. 1:**
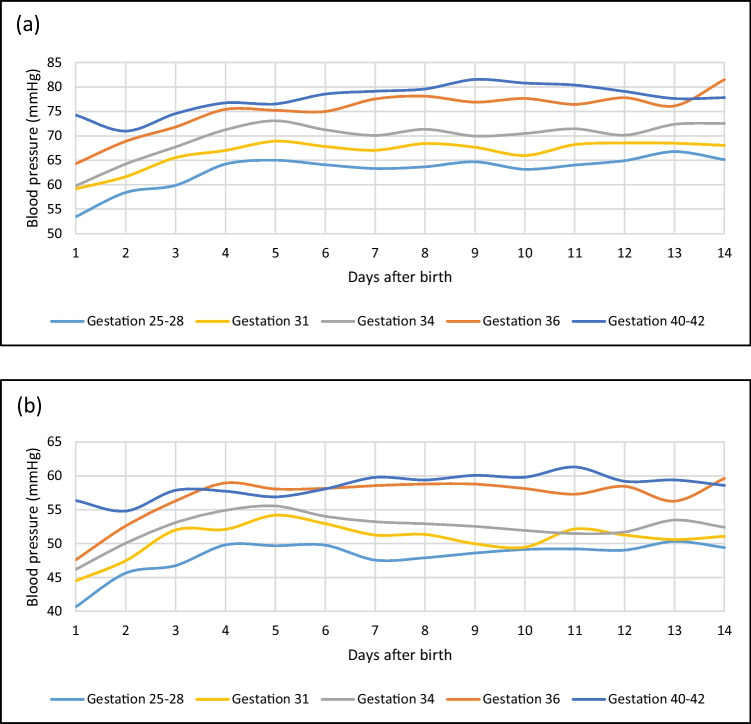
Systolic (**a**) and mean (**b**) blood pressure curves for different gestational ages

**Fig. 2:**
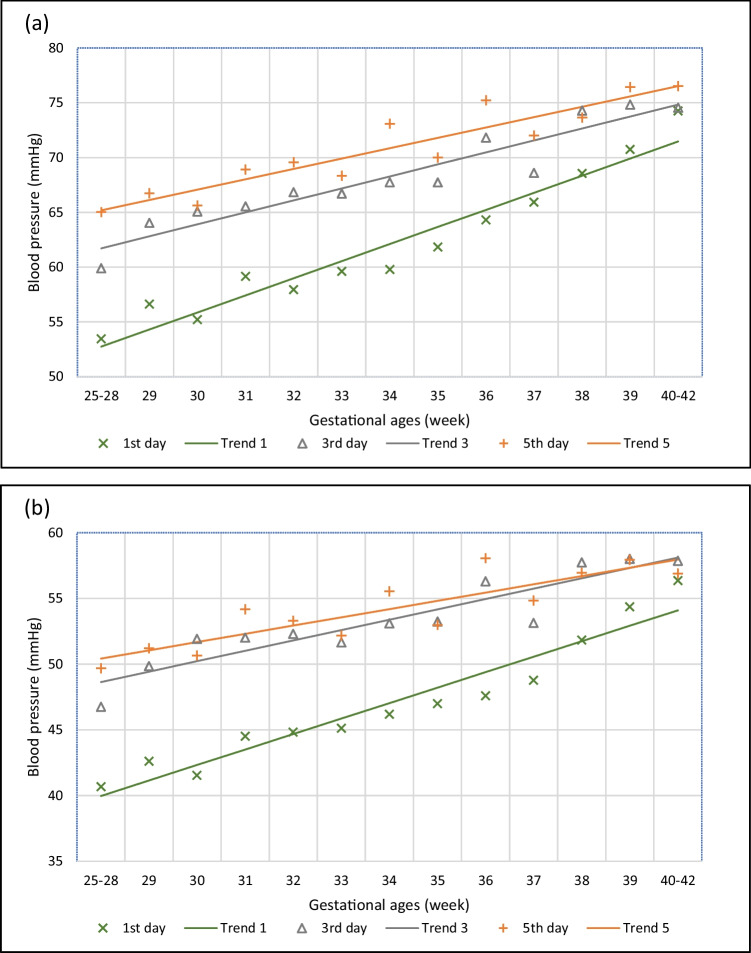
Systolic (**a**) and mean (**b**) blood pressure by gestational ages on the first, third and fifth day of life

**Fig. 3:**
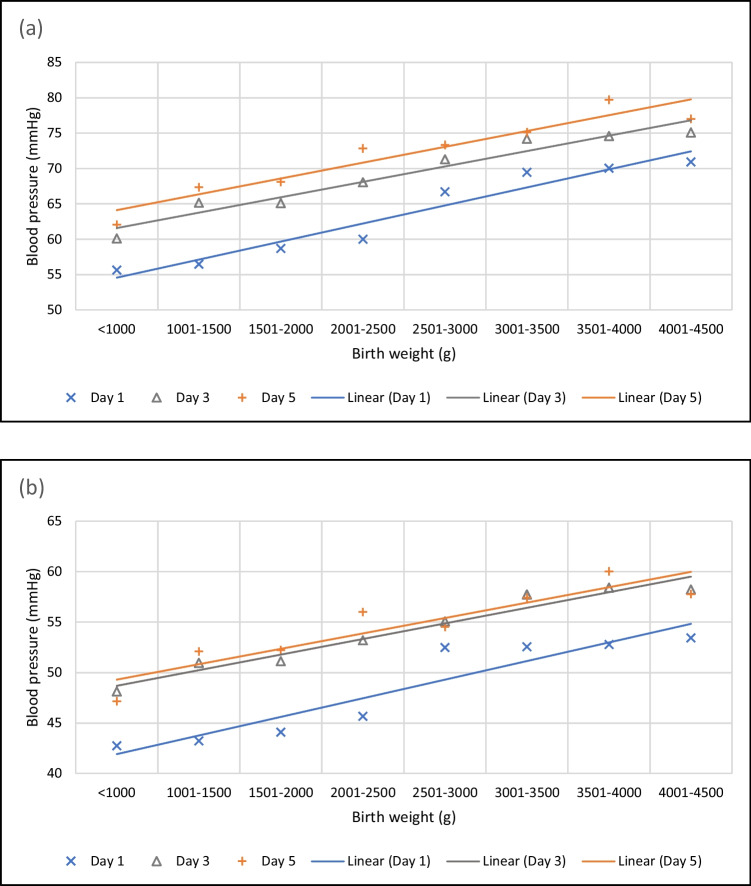
Systolic (**a**) and mean (**b**) blood pressure by birth weight

The unit used the GE Dash 3000 multiparameter monitor system with the GE DINAMAP blood pressure algorithm for blood pressure measurements [19]. The ICCA hospital administration system automatically stored the monitoring data in databases. All non-invasive blood pressure values in the records of the ICCA’s database were retrieved using relevant SQL queries and presented as CSV text files. After a data validity check, the text file with the data was transferred to an SQL database for further analyses in the study. We developed a standalone software PDAnalyzer to perform all the main calculations based on the measured numeric blood pressure values and relevant additional raw data in our database. This data management method prevented data quality problems and maintained data reliability and accuracy. Altogether, 360,507 systolic, diastolic and mean blood pressure data were analysed. The PDAnalyser program calculated the daily average blood pressure values, the corresponding deviations and the daily percentile values. The blood pressure values of the first 2 weeks of life (see Fig. 1) were used for detailed calculations and statistical analyses. The results of the first 5 days have been presented with additional details (see Figs. 2 and 3 and Table 2).

Statistical analysis, as required, was carried out using the IBM SPSS Statistics program (version 29) and Microsoft Excel Data Analysis module. One-sample Kolmogorov–Smirnov test was performed to prove the normal distribution of the data. For the verification, the significance level was set by *p* < 0.05. Linear regression analyses were used to determine trend lines to approximate the arithmetic means calculated from the raw blood pressure values. The graphs were created by Microsoft Excel version 2209.

The Local Human Research Ethics committee approved the study.

## Results

We admitted 839 patients in our neonatal intensive care unit over 3 years (2019–2021). From the total 839 patients, we excluded 136 patients who required inotropic support and mechanical ventilation longer than 24 h, 27 patients who died and 28 patients for other reasons (e.g. diagnosed hypertension, withdrawal syndrome, genetic problems, hydrocephalus). Another 19 patients were excluded due to technical reasons (e.g. insufficient data). After applying the exclusion criteria, we included data from 629 infants in the study, of whom 378 (60%) were preterm and 251 (40%) were full-term patients (see the corresponding gestational age groups in Supplementary Table 1).

We also collected data on the medical conditions (hypertension, diabetes, smoking) of 594 affected mothers. Among them, 35% (*n* = 207) had one of the abovementioned medical conditions, but only 3.9% (*n* = 23) had two or three investigated medical problems together. The maternal medical information was not processed with enough details to let us draw a statistically valid conclusion on their effects on the newborn’s blood pressure.

The patients’ demographic data and clinical characteristics are presented in the following table (Table 1) and a complementary table in Supplementary Table 1.

**Table 1:** Neonatal and maternal clinical characteristics and demographic data

	All patients	25–28 GW	29–32 GW	33–36 GW	37–42 GW
Number of patients	629	27	150	201	251
Male/female patients	380/249	15/12	88/62	124/77	153/98
Average birth weight (g)	2507	1013	1640	2275	3358
Birth weight range (g)	460–5340	460–1520	840–2710	840–2710	1190–5340
Birth weight	Number of patients				
Percentile < 3	36 (6%)	2	2	6	26
Percentile 3–10	47 (7%)	3	9	18	17
Percentile 10–90	479 (76%)	19	127	164	166
Percentile > 90	69 (11%)	3	12	13	41
Average Apgar scores					
Apgar 1 min (SD)	7.59 (± 2.0)	6.3 (± 1.8)	7.0 (± 1.8)	7.4 (± 1.7)	8.3 (± 2.1)
Apgar 5 min (SD)	8.87 (± 1.3)	7.8 (± 1.6)	8.6 (± 1.2)	8.8 (± 1.1)	9.2 (± 1.5)
Neonatal therapy	Number of patients				
Diuretics	40 (6.3%)	9	12	7	12
Analgesia	118 (19%)	3	33	28	57
Caffeine citrate	244 (39%)	27	138	73	5
Ibuprofen	17 (2.6%)	5	8	3	1
Ventilatory therapy (< 24 h)					
Number of patients	40 (6.3%)	5	16	5	14
Length of ventilation (h)	3.3	2.0	2.9	3	5.3
Non-invasive ventilation					
Number of patients	375 (59%)	27	141	136	70
Length of ventilation (d)	5.74	26.5	6.55	3.2	2.18
Maternal data					
Number of mothers	594				
Hypertension	91 (15.3%)	7	20	41	23
IDDM/GDM	82 (13.8%)	2	24	30	26
Smoking	34 (5.7%)	5	11	10	8
Incomplete data	44 (7.4%)	1	7	11	25
Antenatal steroid	Patients	25–28 GW	29–32 GW	33–34 GW	
Incomplete course	82 (27%)	8	37	37	
Complete course	170 (55%)	15	86	69	
Not received	54 (18%)	4	27	23	

*GW* gestational week, *SD* standard deviation, *IDDM* insulin-dependent diabetes mellitus, *GDM* gestational diabetes mellitus

The electronic patient records over the 3 years consisted of 119,714 systolic, 119,700 diastolic and 121,093 mean blood pressure data (i.e. altogether, 360,507 data points). After applying the exclusion criteria, the remaining 629 patients had 44,990 systolic, 44,977 diastolic and 44,971 mean blood pressure values (i.e. altogether, 134,938 data points). In this patient group, the average daily measurement count for one patient was 4.44, and the daily measurement numbers varied between 1 and 12. One-sample Kolmogorov–Smirnov test has confirmed that the blood pressure data sample follows normal distribution.

### Antenatal steroids

We compared the average blood pressure values of the preterm patients (gestational age: 25–34 weeks) who received a complete course of antenatal steroids (*n* = 170, 55%) to the patient group who had an incomplete antenatal steroid course (*n* = 82, 27%) or did not receive steroid prophylaxis (*n* = 54, 18%). A possibly significant difference between the groups above was refused because the applied paired sample *T*-test calculated significance as *p* = 0.191.

### Average blood pressure values of the different gestational age groups in the first 14 days of life

The arithmetical average blood pressure values were determined in different gestational age groups in the first 2 weeks of life. Within the first 14 days of life, 63% of the total measurements (28,456 systolic, 28,444 diastolic and 28,537 mean blood pressure data points) were processed. The patient and measurement numbers in the different gestational age groups are presented in Supplementary Table 1.

All the blood pressure curves representative of a certain gestational age were created. We calculated the daily arithmetical average blood pressure and the standard deviation for each gestational age from the daily blood pressure measurements. Due to the low patient number in the very preterm group and the low measurement number in the term group, we investigated gestational weeks between 25–28 and 40–42 together, respectively. As a representative example, we present the daily average systolic and mean blood pressure curves as the function of time for different gestational age groups in the first two weeks of life (see Fig. 1). The diastolic blood pressure curve is displayed in Supplementary Fig. 1.

The standard deviation was also calculated for the different gestational age groups. The average standard deviation for the systolic, diastolic and mean blood pressures are 9.7 mmHg, 7.9 mmHg and 8.5 mmHg, respectively. We found a statistically significant difference among the average blood pressure values of the different gestational age groups using the SPSS program paired-samples *T*-test (*p*-value < 0.001).

We found that blood pressure increases with an increasing number of postnatal days and gestational age (see Fig. 1).

According to our findings, the systolic blood pressure rise over the first 3 days of life is steeper in preterm infants (25–36-week gestation) than in the term infant group. The systolic blood pressure increased by 3.75 mmHg/day in preterm infants and 1.60 mmHg/day in the term infants in the first 3 days. After the first 5 days, the increasing average systolic blood pressure values might show a mild decrease (see Fig. 1), but the trend remains increasing within the first 2 weeks of life. We experienced a similar pattern examining the diastolic and mean blood pressure data. In the first 3 days, the daily diastolic blood pressure increase was 3.57 mmHg in preterm infants and 1.95 mmHg in term newborns, and the mean blood pressure elevation per day was 3.73 mmHg in the preterm and 1.93 mmHg in the term group. Term infants had a reduction in blood pressure over the first day of life before increasing (see Fig. 1). This indicates the possibility of different physiologic responses between term and preterm infants that need further study.

### Blood pressure in the first 5 days of life by gestational age

As the blood pressure changes are most pronounced in the first week of life, the average daily blood pressure has been calculated on the first, third and fifth days, and the results are graphically presented (see Fig. 2).

Figure 2 shows the systolic and mean blood pressure values and linear regression lines in different gestational ages on the first (Trend 1), third (Trend 3) and fifth day (Trend 5) of life. The diastolic blood pressure graph is presented in Supplementary Fig. 2. According to our analysis, the linear regression lines strongly correlate (systolic: Trend 1 *R*^2^ = 0.9364, Trend 3 *R*^2^ = 0.8975, Trend 5 *R*^2^ = 0.8662, diastolic: Trend 1 *R*^2^ = 0.8483, Trend 3 *R*^2^ = 0.7959, Trend 5 *R*^2^ = 0.6761, mean: Trend 1 *R*^2^ = 0.9244, Trend 3 *R*^2^ = 0.8483, Trend 5 *R*^2^ = 0.7631) with the daily average blood pressure data. The calculated *p* < 0.001 value shows a significant correlation between them.

As shown in Fig. 2, the trend lines increase at a steeper rate in the first day than in the third or fifth day of life. It means the blood pressure difference between the first, third and fifth days of life decreases as the gestational age increases. Based on trend line values, the average rates of change of the systolic blood pressure in the first five days for the 25–28-, 32- and 36-week gestational age groups are 3.11 mmHg/day, 2.49 mmHg/day and 1.88 mmHg/day, respectively. Considering the mean blood pressure, the corresponding values are 2.61 mmHg/day, 2.06 mmHg/day and 1.51 mmHg/day, respectively. Regarding diastolic blood pressure, the corresponding values are 2.30 mmHg/day, 1.79 mmHg/day and 1.28 mmHg/day, respectively.

The average blood pressure of the different gestational age groups increased rapidly over the first 3 days of life (systolic 3.08 mmHg/day, mean 3.17 mmHg/day, diastolic 3.07 mmHg/day). The following 2 days, there was a less pronounced increase in the systolic blood pressure (1.28 mmHg/day) and the mean and the diastolic blood pressures had not increased further (mean 0.41 mmHg/day, diastolic 0.0 mmHg/day).

Supplementary Table 2 provides data on the daily average blood pressure values and percentiles in different gestational age groups on the first, third and fifth day of life.

### Blood pressure by birth weight

We investigated the change in neonatal blood pressure by increasing birth weight and generated eight patient groups; every patient group had a 500 g difference in birth weight. The blood pressure data of 613 patients were included in the analysis. The data of 16 patients with a weight higher than 4500 g was not used for calculation due to the low number of measurements and short hospital stay. We used 18,370 systolic, 18,363 diastolic and 18,439 mean blood pressure data points in the analysis. The systolic and mean blood pressure values and linear regression lines are shown in Fig. 3. The diastolic blood pressure graph is displayed in Supplementary Fig. 3. There is a positive linear correlation between birth weight and blood pressure on the first, third and fifth day of life (see Fig. 3). In the case of systolic blood pressure, the correlation coefficients are R(day1, day3) = 0.968, R(day1, day5) = 0.913 and R(day3, day5) = 0.966. The correlation coefficients calculated for the diastolic blood pressure are R(day1, day3) = 0.880, R(day1, day5) = 0.683, R(day3, day5) = 0.938 and for the mean blood pressure are R(day1, day3) = 0.947, R(day1, day5) = 0.816, R(day3, day5) = 0.946. The blood pressure differences are significant in the different birth weight groups (paired sample *T*-test: significance *p* < 0.001). The systolic blood pressure increased by 8.44 mmHg in the first 5 days. The daily average increment was 2.85 mmHg in the first 3 days and 1.36 mmHg over the following 2 days. The mean blood pressure showed a similar pattern in the first 3 days of life; the calculated average daily increment was 2.86 mmHg, and the blood pressure was almost static over the following 2 days.

The following table (Table 2) contains the average blood pressure values and percentiles of the different birth weight groups on the first, third and fifth days of life.

**Table 2:** Average blood pressure values and 10th, 50th and 90th percentile by birth weight on the first, third and fifth day of life

Birth weight (g)		Day 1 BP				Day 3 BP				Day 5 BP			
		Average	10th Pc	50th Pc	90th Pc	Average	10th Pc	50th Pc	90th Pc	Average	10th Pc	50th Pc	90th Pc
≤ 1000	SBP	56	40	55	72	60	46	59	77	62	49	62	77
	DBP	35	21	36	48	40	27	38	56	39	29	38	51
	MBP	43	27	41	56	48	35	46	64	47	36	47	59
1001–1500	SBP	56	43	55	71	65	53	65	78	67	54	68	79
	DBP	35	24	36	45	42	32	42	53	43	33	43	54
	MBP	43	31	43	56	51	40	51	61	52	41	52	62
1501–2000	SBP	59	48	58	70	65	54	65	77	68	57	68	81
	DBP	35	26	35	44	42	33	41	52	42	32	42	53
	MBP	44	34	44	54	51	42	51	60	52	42	52	64
2001–2500	SBP	60	50	59	72	68	58	68	79	73	60	73	86
	DBP	37	27	36	46	44	35	44	53	45	35	44	57
	MBP	46	36	45	55	53	44	53	62	56	45	55	69
2501–3000	SBP	67	54	64	82	71	59	71	84	73	63	74	83
	DBP	43	30	41	58	45	34	44	56	43	35	42	54
	MBP	52	40	50	68	55	44	54	67	55	45	54	66
3001–3500	SBP	69	56	69	82	74	62	73	87	75	63	75	88
	DBP	42	32	42	52	47	37	46	58	46	37	45	56
	MBP	52	42	52	63	58	46	57	70	57	48	57	68
3501–4000	SBP	70	59	70	81	75	62	74	86	80	69	80	91
	DBP	42	32	41	52	48	37	47	59	48	39	48	59
	MBP	53	43	52	64	58	46	58	70	60	50	60	71
4001–4500	SBP	71	58	70	88	75	62	75	88	77	61	78	90
	DBP	43	35	42	54	47	36	45	59	46	36	44	58
	MBP	53	43	52	66	58	45	58	71	58	45	58	69

*SBP* systolic blood pressure, *DBP* diastolic blood pressure, *MBP* mean blood pressure, *BP* blood pressure, *Pc* percentile

## Discussion

Our single-centre retrospective study provides data on the systolic, diastolic and mean blood pressure of clinically stable neonates. We collected data from a haemodynamically stable term and preterm patient group without substantial hypotension or hypertension risks. The exclusion criteria described above were applied after collecting all the diagnoses and treatment data of the patients.

The study has some limitations, coming from its retrospective nature. The infants’ wakefulness state and the place of measurement are not documented in our electronic records. However, data are collected in natural life circumstances, where the schedule and the quality of the measurements are ensured by following the NICU’s corresponding protocols.

Some therapeutic interventions have uncertain effects on blood pressure (e.g. caffeine, nasal CPAP), but they are part of the routine neonatal treatment; therefore, patients receiving them could not have been excluded from the study. We also could not further divide the patient groups based on non-invasive ventilation data because it would result in several groups with insufficient patients and blood pressure data for a statistical valuation.

Both our patient number and the volume of our measured data are comparable to the largest previous investigations done in this field of research. Most of the other clinical studies investigated healthy patient populations by excluding cardiovascularly compromised patients, but infants with a risk of hypertension were just partly excluded [3, 6]. The Philadelphia neonatal blood pressure study is one of the most extensive investigations to determine normal neonatal blood pressure [8]. Zubrow and his associates involved 608 NICU patients without selection in their prospective multicentre study. They found a strong correlation between blood pressure and gestational age. However, they found that other diagnosis and treatment variables had only a small influence on blood pressure variance. Since earlier research work [3–11], several improvements have arisen in neonatal therapy, and patient survival has improved. These changes also increased the importance of performing new studies and investigating a patient population without severe morbidities and major therapeutic interventions. To meet this target, we omitted patients from our study corresponding to the criteria described above. Parts of the exclusion criteria used in some other research work [6] cannot be applied currently because they may not be appropriate in current clinical practice.

We compared our results to previous studies by using oscillometric blood pressure measurement methods. Kent and colleagues investigated a homogenous preterm patient group without ventilated patients or patients needing inotropic support [3]. Their published blood pressure values by gestational age are close to our results on the second day of life. They also provided blood pressure percentiles by birth weight, similar to our calculated average blood pressure findings. Another study led by Pejovic also published data to estimate normal blood pressure values in neonates [6]. As a comparison, our patient group’s blood pressure values were consequently higher than Pejovic’s findings. We assumed that the difference might be caused by the different patient populations and oscillometric devices.

In clinical practice, average blood pressure values and percentile are helpful. A corresponding table with our results is presented in this paper (see Table 2 and Supplementary Table 2). Previous publications have not presented a similar percentile and average blood pressure table for the first days of life when significant changes in blood pressure occur. We also calculated trend line values (see Fig. 2) that might replace daily average blood pressure results in practice and be used to estimate expected blood pressure results in gestational age groups outside of the presented time interval.

The blood pressure stabilising effect of antenatal steroids is still controversial. Some studies show that antenatal steroid increases neonatal blood pressure [21, 22]. However, another study has not found a significant difference between those who received antenatal steroids and those who did not [20]. Our study shows no essential difference between the complete antenatal steroid group and the group of patients with incomplete or no steroid administration.

Although several studies [4–8] investigate normal blood pressure data on newborns, further research is still needed on the maternal and neonatal therapeutic factors and diagnoses affecting blood pressure. In the future, we plan to compare our current results with the patient group who received inotropic support and add follow-up data. We may collect data on maternal health conditions and medications and study their impact on neonatal blood pressure. We also would like to investigate the effect of antenatal steroids on the entire patient group.

## Conclusion

The main result of our study is that we have calculated the normal average blood pressure by gestational age and birth weight for preterm and term infants for their first 2 weeks of life. Our findings prove that gestational age significantly predicts blood pressure in the first 14 days of life. Our study provides additional data on how blood pressure varies with gestational age and birth weight. The blood pressure tables (see Table 2 and Supplementary Table 2) show that the 10th, 50th and 90th percentiles in the first 5 days of life could be helpful in clinical practice.

## Supplementary information

Below is the link to the electronic supplementary material.

Graphical abstract (PPTX 52 KB)

Supplementary file 1 Information on the gestational age groups, the measurement numbers (see Supplement Table 1) and figures on diastolic blood pressure are presented in Supplementary Table 1 and Supplementary Figures 1, 2, and 3. We also present the average blood pressure and percentile table in the first five days of life (Supplementary Table 2) (PDF 154 KB)

## Data Availability

Data are available on request from the authors.
