# Supplementary material for: Oscillometric arterial blood pressure in haemodynamically stable neonates in the first 2 weeks of life
Source: Pediatr Nephrol. 2023 May 5;38(10):3369–78. doi: 10.1007/s00467-023-05979-x (PMC10465666; doi:10.1007/s00467-023-05979-x)
Supplement: Supplementary file 1 — Graphical abstract (PPTX 52 KB) [file 467_2023_5979_MOESM1_ESM.pptx]

## Slide 1
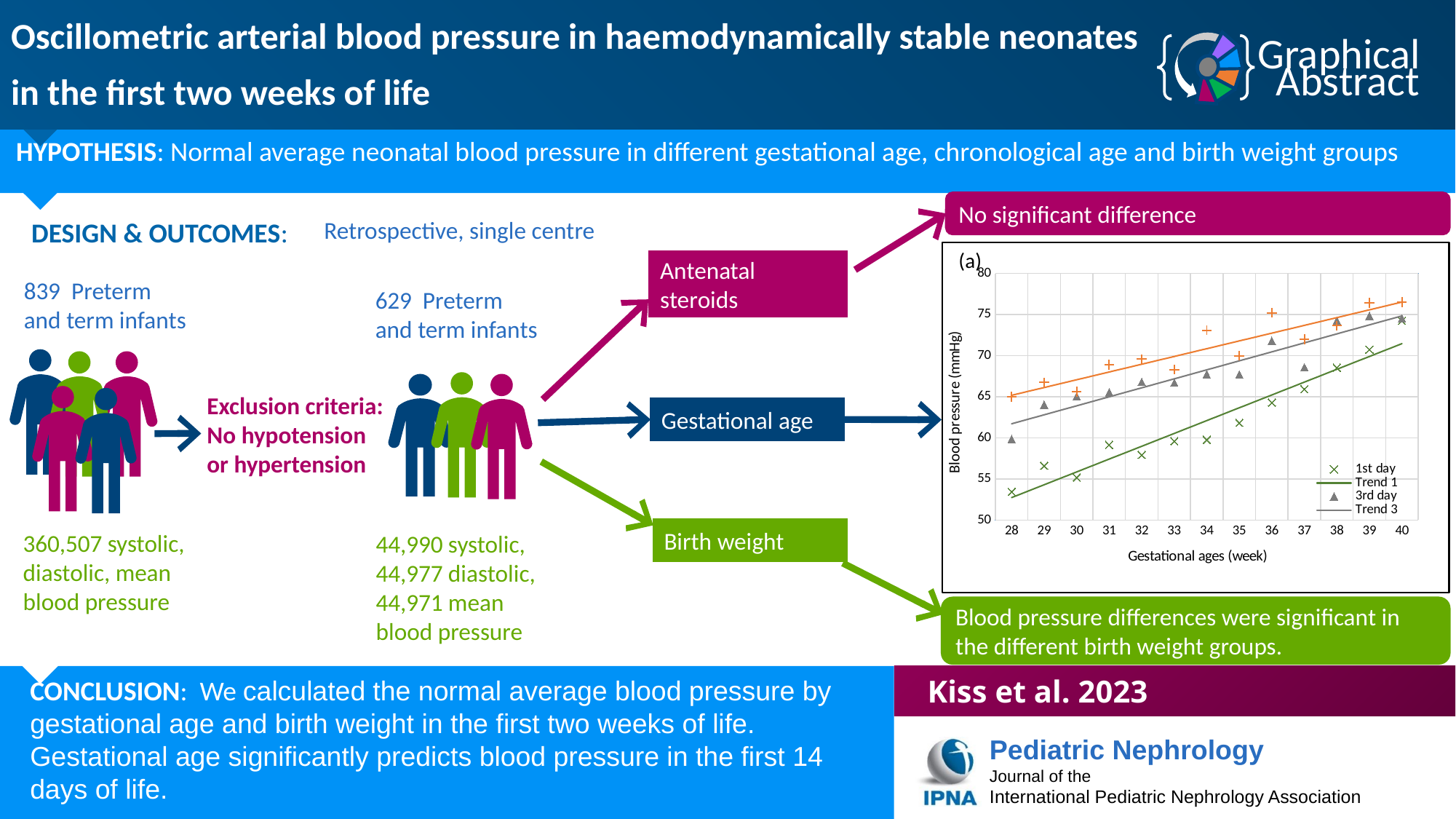

Oscillometric arterial blood pressure in haemodynamically stable neonates
in the first two weeks of life
HYPOTHESIS: Normal average neonatal blood pressure in different gestational age, chronological age and birth weight groups
No significant difference
Retrospective, single centre
DESIGN & OUTCOMES:
### Chart: (a)
| Category | 1st day | Trend 1 | 3rd day | Trend 3 | 5th day | Trend 5 |
|---|---|---|---|---|---|---|
| 28 | 53.45 | 52.7388 | 59.88 | 61.7159 | 65.01 | 65.18130000000001 |
| 29 | 56.62 | 54.2996 | 64.03 | 62.8098 | 66.74 | 66.12660000000001 |
| 30 | 55.19 | 55.8604 | 65.07 | 63.9037 | 65.61 | 67.0719 |
| 31 | 59.16 | 57.4212 | 65.55 | 64.9976 | 68.91 | 68.0172 |
| 32 | 57.94 | 58.982 | 66.83 | 66.0915 | 69.57 | 68.9625 |
| 33 | 59.61 | 60.5428 | 66.71 | 67.1854 | 68.32 | 69.90780000000001 |
| 34 | 59.78 | 62.1036 | 67.74 | 68.2793 | 73.09 | 70.85310000000001 |
| 35 | 61.84 | 63.6644 | 67.72 | 69.3732 | 69.99 | 71.7984 |
| 36 | 64.3 | 65.2252 | 71.82 | 70.4671 | 75.22 | 72.7437 |
| 37 | 65.93 | 66.786 | 68.6 | 71.561 | 72.01 | 73.68900000000001 |
| 38 | 68.54 | 68.3468 | 74.29 | 72.6549 | 73.66 | 74.63430000000001 |
| 39 | 70.74 | 69.9076 | 74.84 | 73.7488 | 76.43 | 75.5796 |
| 40 | 74.25 | 71.4684 | 74.55 | 74.84270000000001 | 76.53 | 76.5249 |Antenatal steroids
839 Preterm and term infants
629 Preterm and term infants
Exclusion criteria:
No hypotension or hypertension
Gestational age
Birth weight
360,507 systolic, diastolic, mean blood pressure
44,990 systolic, 44,977 diastolic, 44,971 mean blood pressure
Blood pressure differences were significant in the different birth weight groups.
Kiss et al. 2023
CONCLUSION: We calculated the normal average blood pressure by gestational age and birth weight in the first two weeks of life. Gestational age significantly predicts blood pressure in the first 14 days of life.
