# Supplementary material for: Oscillometric arterial blood pressure in haemodynamically stable neonates in the first 2 weeks of life
Source: Pediatr Nephrol. 2023 May 5;38(10):3369–78. doi: 10.1007/s00467-023-05979-x (PMC10465666; doi:10.1007/s00467-023-05979-x)
Supplement: Supplementary file 2 — Supplementary file 1 Information on the gestational age groups, the measurement numbers (see Supplement Table 1) and figures on diastolic blood pressure are presented in Supplementary Table 1 and Supplementary Figures 1, 2, and 3. We also present the average blood pressure and percentile table in the first five days of life (Supplementary Table 2) (PDF 154 KB) [file 467_2023_5979_MOESM2_ESM.pdf]

**Information page**	
Article title:	Oscillometric arterial blood pressure in haemodynamically stable neonates in the first two weeks of life
Journal name:	Pediatric Nephrology
Corresponding author:	Judit Klára Kiss
Affiliation:	Department of Paediatrics, University of Szeged, Hungary
Email:	kiss.judit.klara@med.u-szeged.hu
Author names:	Judit Klára Kiss, Anna Gajda, Judit Mari, Judit Németh, Csaba Bereczki

Supplement Table 1 Patient and measurement numbers in the different gestational age groups.

Gestational week	Number of patients	Percentage (%)	Number of measurements within the first 14 days of life		
			Systolic	Diastolic	Mean
25-28	27	4.3	2435	2435	2462
29	27	4.3	1916	1914	1930
30	27	4.3	1878	1878	1889
31	37	5.9	2309	2306	2312
32	59	9.4	3087	3087	3094
33	76	12.1	3252	3251	3269
34	53	8.4	2588	2588	2588
35	38	6.0	1746	1745	1745
36	34	5.4	1073	1073	1076
37	44	7.0	1628	1625	1628
38	49	7.8	1617	1617	1617
39	65	10.3	1974	1972	1972
40-42	93	14.8	2953	2953	2955
Total	629	100	28456	28444	28537

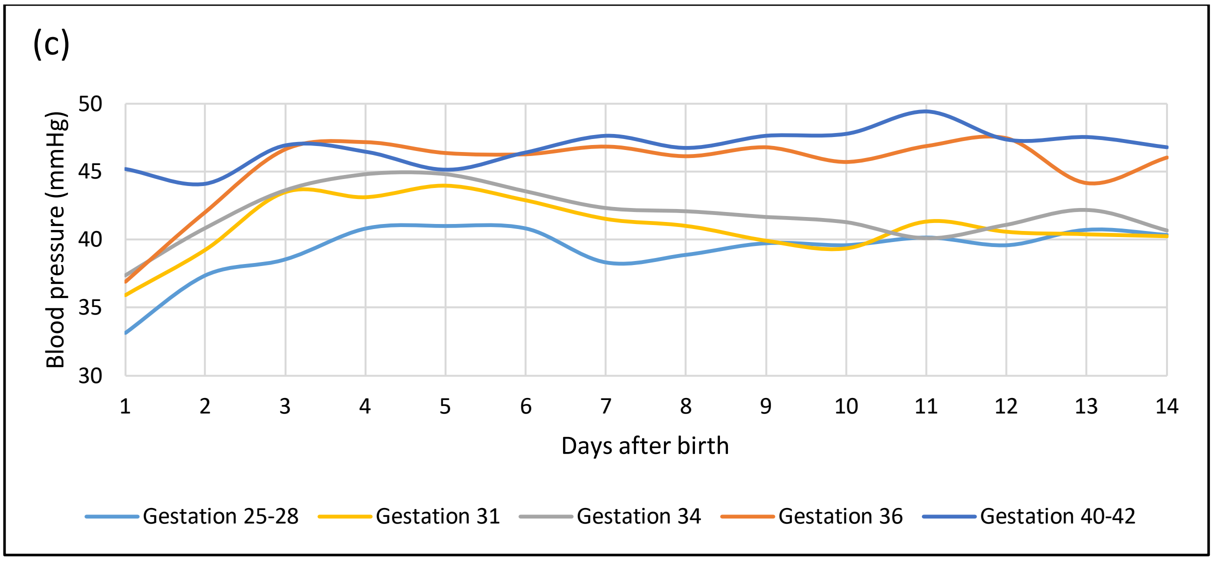

Supplement Fig. 1 Diastolic (c) blood pressure curves for different gestational ages

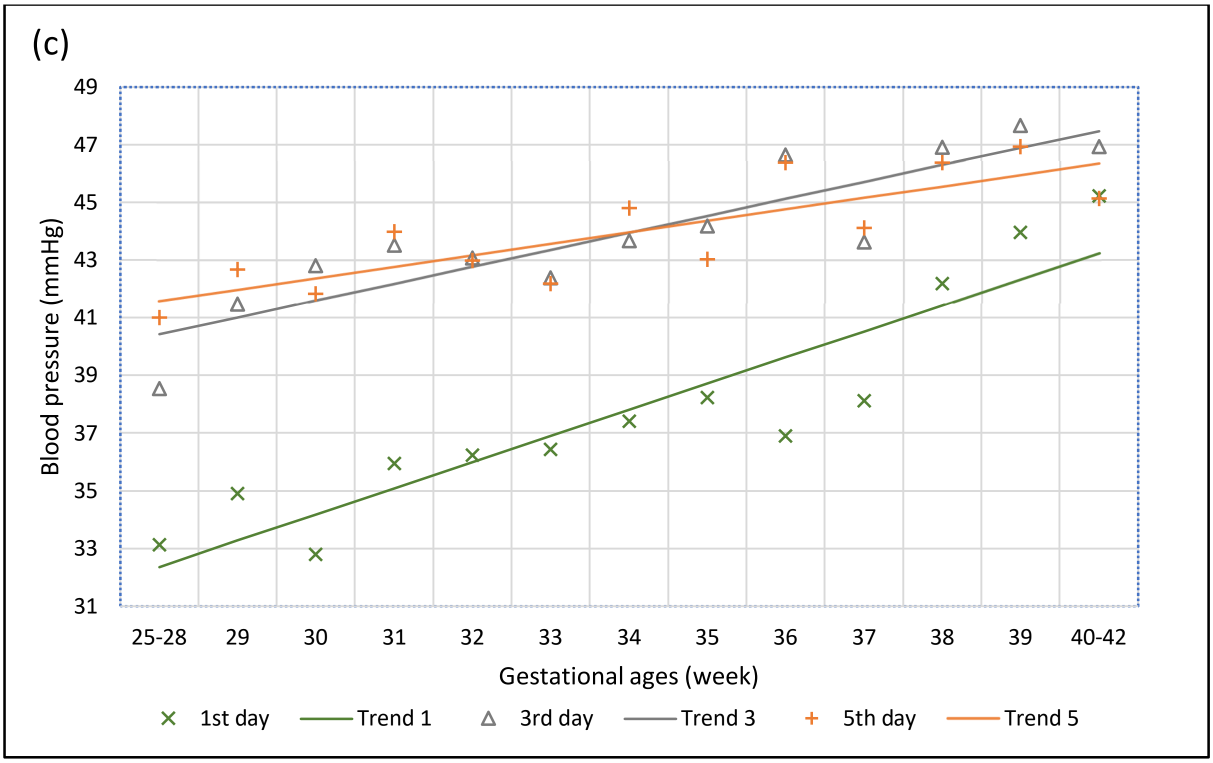

Supplement Fig. 2 Diastolic (c) blood pressure by gestational ages on the first, third and fifth day of life.

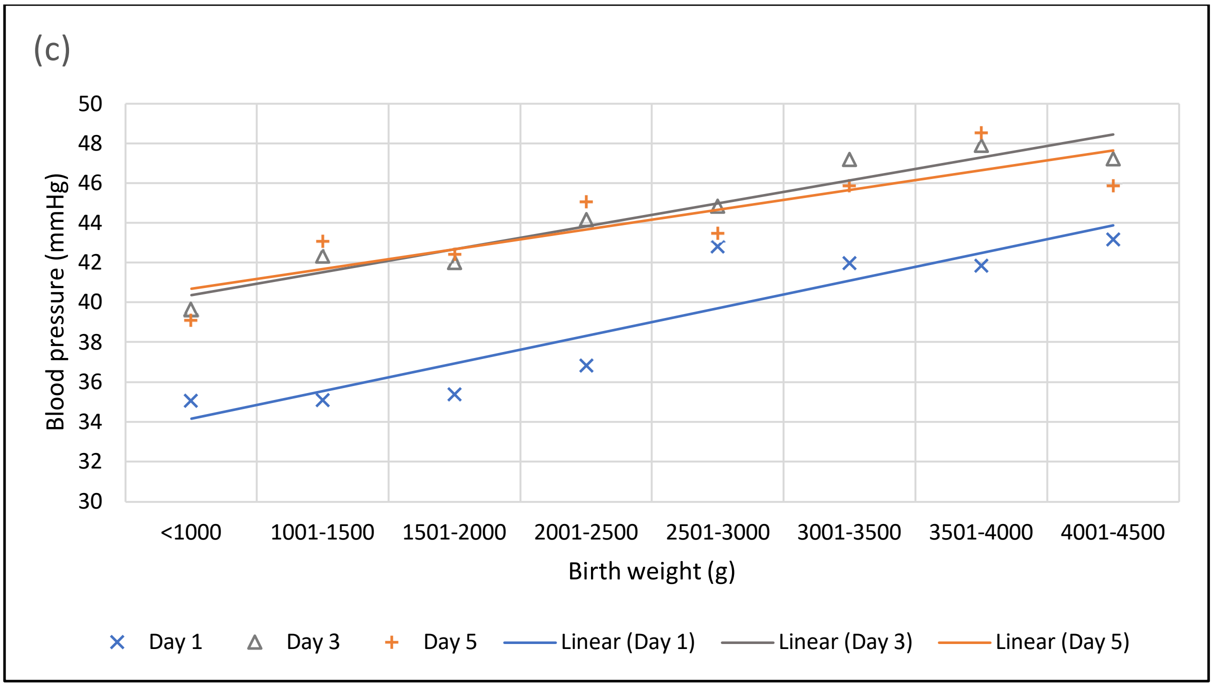

Supplement Fig.3 Diastolic (c) blood pressure by birth weight

Supplement Table 2 Average blood pressure values and 10th, 50th and 90th percentiles on the first, third and fifth day of life

Gestational age		Day 1 BP				Day 3 BP				Day 5 BP			
		Average	10th Pc	50th Pc	90th Pc	Average	10th Pc	50th Pc	90th Pc	Average	10th Pc	50th Pc	90th Pc
**25-28**	SBP	53	40	54	66	60	47	59	73	65	52	64	80
	DBP	33	21	33	45	39	28	38	50	41	31	41	52
	MBP	41	28	40	53	47	36	46	58	50	38	50	61
**29-30**	SBP	56	43	55	69	65	53	64	78	66	54	67	77
	DBP	34	24	34	44	42	31	42	52	42	32	42	54
	MBP	42	31	41	53	51	40	50	62	51	40	52	61
**31-32**	SBP	58	48	58	72	66	54	66	78	69	56	70	83
	DBP	36	27	36	46	43	34	43	53	43	34	43	54
	MBP	45	34	44	56	52	43	52	62	54	42	53	65
**33-34**	SBP	60	50	59	70	67	57	67	78	71	58	70	85
	DBP	37	27	37	46	43	34	43	52	43	33	43	55
	MBP	45	36	45	55	52	43	52	62	54	43	53	67
**35-36**	SBP	63	52	62	75	69	56	68	84	72	60	72	86
	DBP	38	29	38	47	45	35	44	56	44	34	44	56
	MBP	47	37	47	58	54	44	54	67	55	44	55	67
**37-39**	SBP	68	57	67	81	73	60	72	85	74	64	74	87
	DBP	41	31	41	51	46	36	45	57	46	36	45	57
	MBP	52	42	51	63	56	45	56	68	57	45	56	68
**40-42**	SBP	74	61	73	90	75	62	74	87	77	65	76	90
	DBP	45	33	43	62	47	35	46	59	45	37	44	57
	MBP	56	44	54	73	58	45	58	70	57	47	56	68

SBP: Systolic Blood Pressure, DBP: Diastolic Blood Pressure, MBP: Mean Blood Pressure, BP: Blood pressure, Pc: percentile
